# Pivotal role of long non-coding RNA in the development of new therapeutic approaches for plural mesothelioma

**DOI:** 10.1007/s12672-026-04677-y

**Published:** 2026-03-12

**Authors:** Caroline Joseph Kiriacos, Dina M. Elkahwagy, Manar Mansour

**Affiliations:** 1 Pharmaceutical Biology and Microbiology Department, Faculty of Pharmacy and Biotechnology, The German University in Cairo (GUC), Main Entrance Fifth Settlement, Cairo, Egypt; 2 Microbiology and Immunology Department, Faculty of Pharmacy and Biotechnology, German University in Cairo, Cairo, Egypt; 3 Pharmaceutical Biology Department, Faculty of Pharmacy and Biotechnology, German University in Cairo, Cairo, Egypt

## Abstract

Pleural mesothelioma is a rare aggressive tumor that affects the parietal layer of the pleura. It constitutes 90% of mesotheliomas. Usually, the diagnosis takes place 30 to 40 years after asbestos exposure. Thus, patients are diagnosed at a very late stage, leading to a poor prognosis, with a low median survival rate of approximately one year and a 5-year survival rate of only 5%. The available treatment usually consists of a multimodal approach, including chemotherapy (platinum-based drugs and antifolates), surgery, and radiotherapy. Yet, new therapeutic approaches are still needed to achieve higher survival rates. Long non-coding RNAs are transcripts of more than 200 nucleotides and have been proven to participate in various cellular processes and biological mechanisms. Upon dysregulation, many biological pathways can be affected, initiating cancers or even promoting their aggressiveness. These non-coding RNAs have been studied for their therapeutic potential in various cancers. The role of long non-coding RNAs in PM, whether in regulating pathogenicity or as potential therapeutic targets, requires further investigation. This review highlights the role of lncRNAs in PM pathogenesis and their potential as therapeutic targets.

## Introduction

**Fig. 1:**
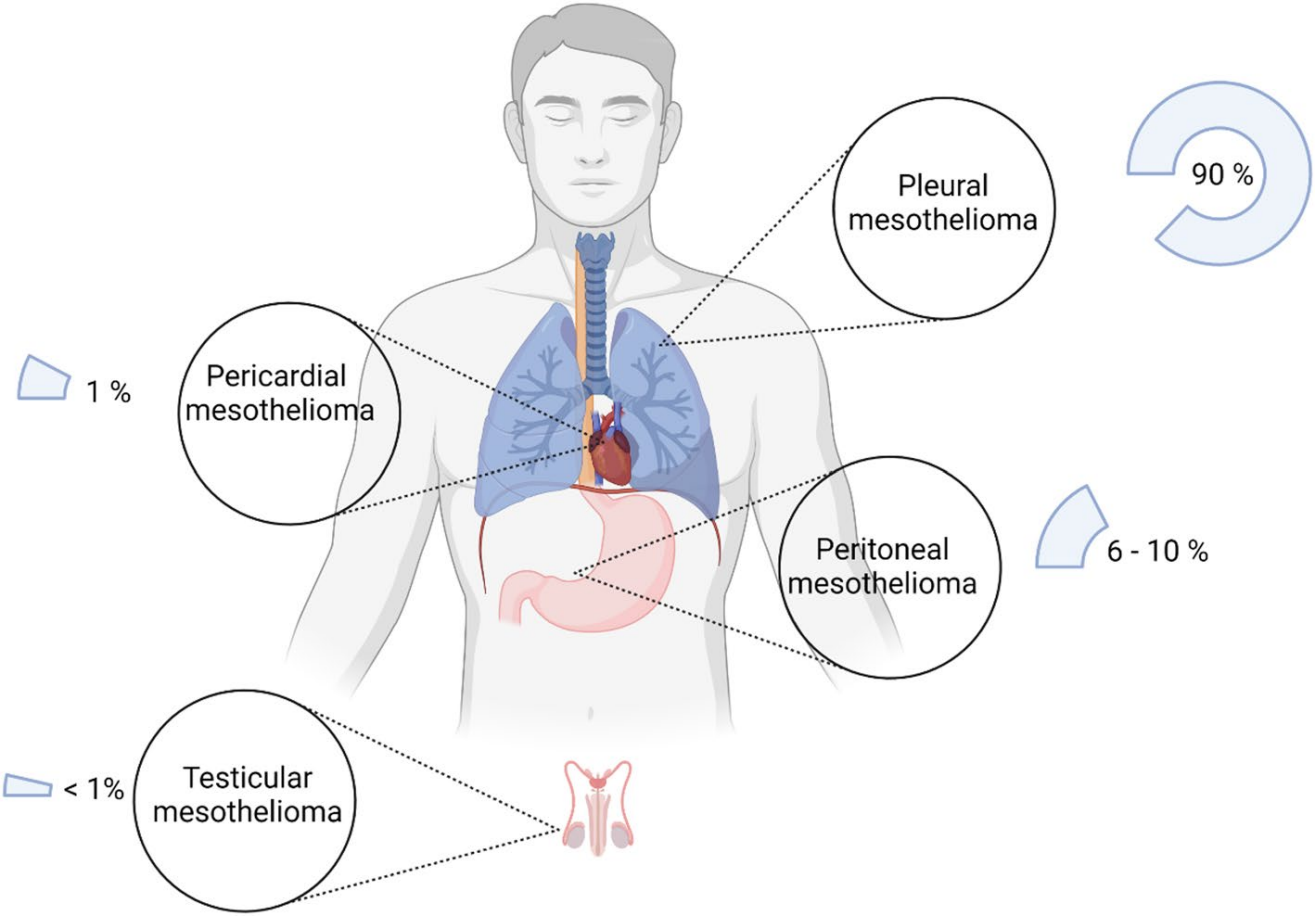
Types of mesothelioma and their corresponding percentages. The four types of mesothelioma are demonstrated with their percentages of occurrence. Pleural mesothelioma is the predominant type, accounting for 90% of cases. Peritoneal mesothelioma constitutes 6–10% of cases. Pericardial and Testicular mesotheliomas had the lowest percentages, with 1% and less than 1%, respectively

 Mesothelioma, a highly aggressive rare tumor, targets the serosal surface, specifically the mesothelial lining of the pericardium, peritoneum, tunica vaginalis, and pleura [1]. Pleural mesothelioma (PM) is the most prevalent, accounting for over 90% of cases [2, 3]. Previously, the WHO classified this as malignant pleural mesothelioma. However, in 2021, the WHO dropped the prefix ‘malignant as all mesotheliomas are by default considered malignant, whether localized or diffuse [4]. Figure 1 [5, 6] highlights the different types of mesothelioma and their respective percentages.

Pleural mesothelioma comprises morphological differentiation; therefore, it is divided into three histologic subtypes: epithelioid, sarcomatoid, and biphasic [7]. Although any subtype of this cancer is aggressive, the epithelioid subtype has a better prognosis than the other subtypes [8]. Sarcomatoid holds the worst prognosis [8, 9].

The primary factor contributing to the aggressiveness of pleural mesothelioma is the latency period of the disease. Diagnosis typically occurs 30–40 years after asbestos exposure, leading to diagnosis at an advanced stage [10]. Moreover, the symptoms that emerge after this latent period are non-specific to the disease, exacerbating the challenge of combating it. These symptoms commonly include dyspnea, pleural effusion, and chest pain [11]. The median survival is 9–12 months, and there is only a 5% chance of 5-year survival, which serves as proof of its aggressiveness [12].

### Treatment

The available treatment options are few; however, the multimodal approach consisting of chemotherapy, radiotherapy, and surgery remains the standard of care, aiming to increase survival and improve quality of life [13]. For chemotherapy, a combination of platinum-based drugs (cisplatin or carboplatin) and antifolates (pemetrexed or raltitrexed) is used in the first and second lines of treatment, before or after surgery, or even in cases of unresectable tumors. However, chemoresistance remains a significant challenge [14]. Recent advancements in immunotherapy have changed this landscape.

Immunotherapy as immune checkpoint inhibitors (ICIs) have shown improved overall survival compared with chemotherapy. The combination of nivolumab and ipilimumab is now considered the first-line of treatment for unresectable PM tumors, following promising clinical results [15, 16]. This recommendation is based on the results of the Phase III CheckMate 743 trial and led to FDA approved in October 2020 for first-line use in advanced or unresectable pleural mesothelioma [17–19].

The dual blockade of PD-1 (nivolumab) and CTLA-4 (ipilimumab) is thought to have a synergistic effect, potentially leading to better outcomes, particularly in tumors with low PD-L1 expression. However, combined immunotherapy is associated with higher toxicity, with nearly half of patients experiencing grade 3 or 4 adverse events, compared to 17% with single-agent therapy [20]. Moreover, it is effective in only a subset of patients and is associated with immune-related adverse events (IRAEs) and higher discontinuation rates [21–23]. Retreatment with nivolumab-ipilimumab after a durable response is feasible but carries a risk of immune-related toxicity [22].

The benefit of first-line immunotherapy is particularly evident in non-epithelioid mesothelioma, where chemotherapy is less effective. In contrast, epithelioid mesothelioma patients, can still achieve durable disease stabilization with chemotherapy, and its efficacy is comparable to immunotherapy in this subgroup [24].

However, critical analyses and comparisons with RWD tend to consistently flag important limitations and potential biases associated with the registrational ICI trials in MPM. While CheckMate 743 did show a statistically significant overall survival benefit with nivolumab plus ipilimumab in the trial population [25], subsequent evidence demonstrated that patient selection might have influenced the magnitude of benefit reported. By enrolling patients with generally favorable prognosis-most having an ECOG performance status of 0–1 and fewer comorbidities-the median survival outcomes exceed those observed in broader, unselected clinical practice cohorts. Both meta-analytic data and RWE studies indicate that real-world patients often present with poorer performance status, higher disease burden, or mixed histological subtypes and, in turn, worse survival outcomes compared with those in controlled trial settings [26–28]. Moreover, the design of earlier supportive studies, including the Phase 2 PrE0505 trial, has been critically discussed because it relies on historical control groups rather than a contemporary, randomized comparator arm, which can inherently inflate the perceived efficacy of the novel regimen [29]. While dual-ICI therapy has shown its greatest benefit in patients with the non-epithelioid subtype, this is often offset by substantially higher rates of Grade 3/4 immune-related adverse events, raising important considerations with respect to the risk-benefit balance in frail or comorbid MPM patients [26–28]. These analyses collectively emphasize the need for caution in interpreting the results from the ICI registrational trials and stress the importance of integrating real-world evidence when applying ICI-based regimens to routine clinical practice.

Additional clinical trials are currently underway to further investigate immunotherapy as a first-line treatment in pleural mesothelioma **(**Table 1**)**.

**Table 1:** Ongoing clinical trials investigating first line immunotherapy in pleural mesothelioma

Trial name/ID	Immunotherapy agents	Control	Patient population	Status	References
DREAM3R (NCT04334759)	Durvalumab + chemotherapy	Nivolumab–ipilimumab	Advanced pleural mesothelioma	Phase 3, ongoing	[224]
BEAT-MESO (NCT03762018)	Atezolizumab + chemotherapy + bevacizumab	Standard chemotherapy + bevacizumab	Advanced pleural mesothelioma	Phase 3, ongoing	[224]
PrE0505	Durvalumab + cisplatin + pemetrexed	Historical controls	Previously untreated pleural mesothelioma	Completed, phase 2	[225, 226]

Surgery is performed either for curing or as a palliative measure to control the symptoms. However, it should be combined with chemotherapy or radiotherapy [30, 31]. Patients with PM who will undergo tumor resection have two options: well-tolerated pleurectomy/decortication (P/D) and more aggressive extrapleural pneumonectomy (EPP) [32]. The choice between both depends on the extent of the disease and the strategy of the used multimodality approach; however, the selection between them is still debatable [33–35].

The main aim of radiotherapy in PM patients is tumor volume reduction and lowering the risk of recurrence after surgery. Intensity-modulated radiotherapy (IMRT) is an advanced technique used to reduce the dose of radiation received by the surrounding organs while maximizing the dose to the tumor to decrease any harm to unintended organs; however, this is not guaranteed. Radiotherapy can be used as a neoadjuvant approach before surgery to reduce tumor volume and microscopic spread of malignant cells, allowing for complete removal of the tumor. It could also be used as an adjuvant in a multimodal approach with chemotherapy and surgery [30]. For patients who are unable to undergo tumor removal surgeries, radiation can be used as a palliative therapy; however, pulmonary toxicity is a risk when curative doses are applied [36].

### Long non-coding RNA

This group of ncRNAs is classified as lncRNAs with a transcript length above 200 nucleotides, which are transcribed by RNA polymerase II, and their translation into proteins is not possible [37]. Their structures are highly heterogeneous, enabling various 3D shapes and conformations, allowing them to interact with different macromolecules [38]. They play a significant role in development and differentiation and epigenetic regulation of gene expression by chromatin modification. Moreover, they act as sponges for miRNAs, suppressing their activity. Their regulatory mechanisms are complex and depend on their position; they can act by targeting mRNAs or proteins as post-transcriptional regulators or bind directly to DNA or transcription factors for regulation at the transcriptional level [38, 39]. Different functions of lncRNAs were reported They could function as decoys, guides, signals, scaffolds or miRNA precursor. Those mechanisms can take place in a parallel way [40].

The advent of next generation sequencing technology has brought to the forefront a multitude of lncRNAs exhibiting abnormalities in various cancerous diseases. They are crucial for normal homeostasis and so can ultimately pose threats of disease upon deviation in their levels [41, 42]. As highlighted by numerous research papers, deregulation of lncRNAs is associated with the onset, progression or even orchestration of different cancer hallmarks [43–48].

As discussed before, the aggressiveness of PM remains a significant barrier in its treatment, with very low survival rates. New treatment modalities are needed. LncRNAs are paving their way in the field of therapeutics. This review highlights the most recently discussed lncRNAs in PM, and evaluate their potential as therapeutic targets.

### Long non-coding RNA in PM

#### SNHG8

Overexpression of SNHG8 has been reported in many cancers, including colorectal cancer [49, 50], breast cancer [51], and lung cancer [52]. In PM, it was found to be upregulated in both cell-lines and tissues [53]. The aberrantly expressed lncRNA has been associated with numerous oncogenic characteristics, including cell proliferation and autophagy [51, 54–57], migration and apoptosis [51, 58–60]. Knockdown of SNHG8 has been shown to increase apoptosis in cancer cell lines [51, 52, 60–62]. The lncRNA was found to have diverse interaction partners such as the enhancer of zeste homolog 2 (EZH2) [60], is enzymatic catalytic subunit of polycomb repressive complex 2 (PRC2) that can alter downstream target genes expression by trimethylation of Lys-27 in histone 3 (H3K27me3). Targeting EZH2 for cancer therapy is a hot research topic now and different types of EZH2 inhibitors have been developed. It has also been reported that it interacts with miRNAs miR-149, miR-491 [54]. SNHG8 has been upregulated in the plasma of epithelioid PM patients when compared to controls [63] Table 2. Targeting SNHG8 for cancer treatment has been explored in numerous preclinical studies using RNA interference (RNAi) and antisense oligonucleotides (ASOs) to reduce SNHG8 expression in various cancer cell lines and animal models [52]. Reducing SNHG8 levels through siRNA or shRNA has consistently shown inhibitory effects on cancer cell proliferation, migration, invasion, and tumor growth in vitro and in vivo across various cancer types, including NSCLC [52].

**Table 2:** Key LncRNAs implicated in pleural mesothelioma

lncRNA Name	Role in PM	Mechanism of action in PM	References
PVT1	Oncogenic	Targets FOXM1, promotes proliferation and migration	[117]
MALAT1	Oncogenic	Sponges miR-141-3p, upregulates YAP1, promotes proliferation, migration, invasion, and EMT	[63]
GAS5	Tumor Suppressor, Potential Biomarker	Lower expression in PM cells, upregulated upon growth arrest, affects cell proliferation, potential circulating biomarker	[108]
LINC00152	Oncogenic	Cooperates with EZH2 to promote proliferation, migration, and invasion	[66]
SNHG8	Potential Biomarker, Promotes Chemoresistance (in other cancers)	Elevated in plasma of PM patients, potential role in drug resistance	[63]
POT1-AS1	upregulated in both patient cohort and PM cell-lines	Not known yet. More studies are required.	[53]
ZFAS1	Oncogenic	overexpressed in RNA-sequenced data from biphasic or epithelioid PM patients and was found to be upregulated compared to the sarcomatoid subtypereducing the ZFAS1 levels in PM offers a promising therapeutic target.	[59, 60]
CASC2	Tumor suppressor	CASC2 has been reported to exert its suppressive activity through sponging miRNAs the chromosomal region where the gene of the lncRNA is located has been reported to be lost in 37% of human mesothelioma cells obtained from patients	[89–92, 96]
H19	-Oncogenic -Overexpression in PM is associated with worse overall survival. -Induction of cisplatin chemo resistance in lung cancer cell lines	No enough studies were available on H19 and its oncogenic regulatory pathways or its molecular involvement in PM.	[80, 157]
PCAT6	serve as a diagnostic and prognostic biomarker in PM	Associated with overexpression of lysine-specific demethylase 5B (KDM5B)	[80, 166, 170]
EFGR-AS1	Oncogenic	Associated with high resistance to therapy by EGFR tyrosine kinase inhibitor (TYR)	[172, 173]
NEAT1	Oncogenic	It is BAP1 dependent and promotes EMT through regulation of EZH2	[179–181]

#### POT1-AS1

POT1-AS1 is a relatively novel lncRNA with few studies conducted on its nature and association with cancer. This LncRNA was investigated in PM cell-lines and tissues obtained from the EPP of patients versus cryopreserved benign pleura tissues, and was found to be upregulated in both patient cohort and PM cell-lines [53]. Table 2.

In gastric cancer, it was found to act oncogenically as a sponge for miR-497-5p, which in turn elevated pyruvate dehydrogenase kinase 3 (PDK3), whose overexpression is believed to progress carcinogenesis in other cancers. Suggesting that the POT1-AS1/miR-497-5p/PDK3 axis is a potential target in anticancer therapy [64, 65].

#### LINC00152

Long intergenic non-protein coding RNA 00152 (LINC00152), also known as CYTOR, is a lncRNA that is overexpressed in several malignant tumors and is involved in tumorigenesis. 46 High LINC00152 expression has been associated with a poor prognosis in patients with mesothelioma.46.

LINC00152 knockdown inhibited the proliferation, migration and invasion of several mesothelioma cell lines. Its activity is believed to be associated with EZH2, as suppression of the later eliminated the carcinogenic activity observed upon knockdown of the lncRNA [66] Tables 2 and 3. Direct interaction between the two elements has been associated with the progression of various other cancers [67], with the lncRNA further suppressing the expression of the anti-inflammatory/pro-apoptotic cytokine Interleukin-24 (IL-24) in the lung adenocarcinoma (LUAD) subtype of non-small cell lung cancer (NSCLC).

**Table 3:** Preclinical studies targeting LncRNAs in mesothelioma (In Vitro)

lncRNA Target	Targeting strategy	Key findings	References
PVT1	siRNA	Decreased proliferation and migration, increased G2/M phase	[117]
MALAT1	shRNA	Inhibited proliferation, migration, invasion, induced G0/G1 phase arrest	[145]
GAS5	shRNA	Increased expression of glucocorticoid responsive genes, shortened cell cycle	[108]
LINC00152	siRNA	Inhibited proliferation, migration, and invasion	[66]

#### ZFAS1

ZFAS1, also known as ZNFX-AS1, resides on chromosome 20, specifically on band 20q13.13. Elevated expression of ZFAS1 has been observed in cell lines and tissues of many cancers such as Gastric cancer [68, 69], Liver cancer [70], Colorectal cancer [71–73], and nasopharyngeal carcinoma [74]. ZFAS1 was also found to be downregulated in triple negative breast cancer [75] and Hepatocellular carcinoma [76], with decreased levels being tied to increased oncogenesis.

The observed variation in expression levels suggests a potential dual role for the lncRNA, serving both as an oncogene and as a tumor suppressor, possibly through different isoforms.

ZFAS-1 has been shown to have a functional role in the progression of cancer through various pathways. It acts to increase cell proliferation [68, 70, 72, 73, 75, 77], enable migration and invasion of cancerous cells [74, 78, 79], mediate the epithelial-mesenchymal transition (EMT) process [71, 73, 78], and reduce apoptosis [68, 72, 73] in various cancer cell lines.

ZFAS1 was overexpressed in 5% of RNA-sequenced data from biphasic or epithelioid PM patients [80] and was found to be upregulated compared to the sarcomatoid subtype of the disease [81] Table 2.

Experimental assays in one study revealed an increase in ZFAS1 levels in both clinical samples and cell lines obtained from patients with NSCLC. Reducing ZFAS1 levels demonstrated inhibition in the progression of NSCLC cells by suppressing cell proliferation and invasion and enhancing apoptosis. Mechanistic investigations further showed that ZFAS1 promotes NSCLC progression by modulating the miR-150-5p/HMGA2 axis [82]. In another study focused on Lung adenocarcinoma (LUAD), ZFAS1 was also found to be upregulated in both tissue samples and cell lines. It was further confirmed to promote cell proliferation, migration, and invasion by similarly interacting with a miRNA, downregulating miR-1271-5p, which upregulates its direct target fibroblast growth factor receptor substrate 2 (FRS2) [83]. Similarly reducing the ZFAS1 levels in PM offers a promising personalized therapeutic target for biphasic and epithelioid more than sarcomatoid. However, further studies are still needed.

#### CASC2

Cancer susceptibility candidate 2 (CASC2) has been found to be downregulated in many cancers including Glioma [84], endometrial cancer [85], colorectal cancers [86] and lung cancer [87]. Some studies have reported upregulation of the lncRNA, as in the case of Weng et al., where high CASC2 levels were associated with a worse prognosis and overall oncogenesis of astrocytoma [88]. CASC2 has been reported to exert its suppressive activity through sponging miRNAs [89–92] Table 2. One such example is miR-21, which is overexpressed in gliomas and renal cell lines. This dysregulated axis (Downregulated CASC2/Overexpressed miR-21) has been shown to mitigate cell proliferation, migration, invasion and reduce apoptosis in glioma cell lines [84]. Other examples include the miRNA-18a/BTG3 axis studied in CRC cell-lines, wherein inhibited CASC2 positively regulated the miRNA, which downregulates the expression of BTG3, resulting in the increase of cell proliferation, migration, invasion and reduction of apoptosis [93].

In lung cancer, CASC2 expression was reduced in both LUAD tissues and cancer cell lines [94, 95]. Interplay between the lncRNA and miR-4735-3p was associated with the inhibition of cell proliferation and migration through the rapamycin (mTOR) pathway, with a direct correlation between CASC2 expression and the mTOR protein [95]. CASC2 was also found to act through a miR-21/p53 axis. Addition of a mimic increased p53 expression and inhibited the associated carcinogenic phenotypes in the LUAD cell-lines.

One study has reported (through the analysis of RNA-sequencing data of The Cancer Genome Atlas (TCGA) Mesothelioma dataset) that high expression of CASC2 was associated with better Overall Survival (OS), with samples being either biphasic or epithelioid subtypes [80]. Furthermore, the chromosomal region where the gene of the lncRNA is located has been reported to be lost in 37% of human mesothelioma cells obtained from patients [96]. Introducing a copy of the CASC2 gene might offer a potential therapeutic strategy.

#### GAS5

Growth arrest-specific transcript 5 (GAS5), is one of the novel lncRNAs found in certain cancers such as mammary carcinoma, prostate cancer, lung cancer, gastric cancer, and others [97–99]. Chromosome 1q25 harbors GAS5 with 630 nucleotides [100]. Normally, it is important for stopping cell growth and inhibiting cell cycle progression [101]. Its bioactivity was first investigated in human T cells, and results showed an inverse relationship between its expression level and both apoptosis and growth [102]. Other studies noted that its downregulated level in many solid malignancies could be a sign for larger tumor size, advanced stage, and therefore poorer prognosis [98, 103, 104]. This was evident in NSCLC patients as well, in which 72 tissue specimens showed lower expression levels of GAS5 than normal ones [105]. Its downregulation in NSCLC was linked to tumor size and metastasis [103]. Furthermore, its downregulation was linked to drug resistance in lung adenocarcinoma A549 cell line [106]. Its overexpression in osteosarcoma has led to proliferation, migration and suppression of EMT [107].

Regardless, GAS5 RNA levels showed significant overexpression in PM tissues when compared with normal non-cancerous tissues [108], GAS5 expression is upregulated upon growth arrest induced by the inhibition of Hedgehog and PI3K/mTOR signaling in in vitro PM models [108] Table 2. Silencing of GAS5 has been shown to increase the expression of glucocorticoid-responsive genes and shorten the length of the cell cycle [108] Table 3. GAS5 is abundant in quiescent tumor cells and its expression is correlated with podoplanin expression in PM tissue [108]. Notably, circulating GAS5 has been identified as a potential complement marker for the detection of mesothelioma using liquid biopsies [109] Daniel et al. studied the potential of GAS as a diagnostic biomarker for PM. It was verified as an appropriate complementary marker in a panel with calretinin and mesothelin [110].

#### PVT1

Plasmacytoma variant translocation 1, or PVT1, is a lncRNA that has been linked with the rate of aggressiveness for many cancers [111]. For instance, PVT1 is overexpressed in breast cancer and interacts directly with SRY-box2 (SOX2) leading to EMT [112]. It was also found to guide EMT in pancreatic cancer by decreasing levels of the cyclin-dependent kinase p21 [113]. Moreover, PVT1 was shown to drive EMT by regulation of genes epigenetically, potentially through interaction with EZH2 [114–120], thus linking it to EMT and poor prognosis in many cancers [80]. PVT1 has also been implicated in chemotherapy resistance and plays a role in tumor survival [121, 122].

PVT1 location is 8q24.21, the same location for c-Myc, a gene encoding a transcription factor required for regulation of various processes such as proliferation, growth, differentiation and apoptosis [123–125]. It has been shown that when PVT1 expression levels increase, c-Myc levels also increase in the amplified 8q24 region [126]. The cooperation between them has been shown to promote PM, making this amplified region an attractive therapeutic target [127]. The locus of PVT1 compromises a cluster of at least six miRNAs spanning the region, which further adds to its complexity [128, 129].

Upregulation of PVT1 was detected in several mesothelioma cell lines. PVT1 knockdown resulted in downregulation of Forkhead box M1 (FOXM1) expression, as indicated by reduced FOXM1 expression at the protein level. This in turn, caused decreased mesothelioma cell proliferation, migration, and increased the proportion of cells arrested in the G2/M phase, suggesting an impact on cell cycle regulation [130].

PVT1’s interaction with FOXM1 was validated in gastric cancer as well, causing similar pro-oncogenic activities [131]. The lncRNA is widely associated with other cancers, both thoracic and otherwise, with several targets that include proteins such as EZH2 [117] and p53 [132], miRNA such as miR-125 [133] See Tables 2 and 3.

A study carried out by Riquelme et al. aimed to understand the role of PVT1 in PM pathogenesis, highlighted its action as an oncogenic RNA in PM. When PVT1 was knocked down by short interfering RNA (siRNA) in the MSTO-211 H cell line, cisplatin sensitivity increased and cell viability decreased. Another study confirmed the enhancement of cisplatin sensitivity after PVT1 knockdown by inducing pro-apoptotic Bcl-2 like protein 14 (BCL2L14), lymphotoxin beta (LTB), FS ligand (FASLG) and tumor necrosis factor receptor superfamily member 1B (TNFRSF1B). Moreover, anti-apoptotic BCL2L1, ICEBERG (Caspase 1 inhibitor), BCL2 and Baculoviral IAP repeat-containing protein 8 (BIRC8) were suppressed [127].

Its complex relationship with miRNAs was also studied, and it was found that miR-1204 is overexpressed in the MSTO-211 H cell line when PVT1 is upregulated. When miR-1204 is depleted, anti-apoptotic genes are expressed. Thus, PVT1 is suggested to have intricate mechanisms contributing to PM chemotherapeutic resistance and survival [127].

#### MALAT-1

Metastasis-associated lung adenocarcinoma transcript 1, also known as NEAT2 or nuclear enriched abundant transcript 2, is another name for the MALAT1 lncRNA. This lncRNA was first identified in NSCLC as a marker for metastasis and low survival rates [134]. Since then, it has been investigated and shown to be a prominent RNA in various cancer types such as liver, breast, pancreatic, gastric cancers, and others, representing a hallmark for cancer onset and development [135–141]. In lung adenocarcinoma, MALAT1 induces cisplatin resistance by activating STAT3 and inducing the expression of MRP1 and MDR1 [142]. MALAT1 has also been shown to induce angiogenesis, invasion, and proliferation of cancerous cells [143].

In-silico studies for PM samples showed overexpression of this lncRNA, especially in the epithelioid subtype [80]. Another study was conducted to investigate the expression of MALAT1 in PM cell lines, which reported upregulation in its levels in the cancerous lines. After silencing MALAT1 in H226 and H2452 cancerous PM lines, proliferation was inhibited, and cell cycle arrest occurred. These results were confirmed by in-vivo study on mice, where MALAT1 knockdown was highly associated with tumor size and growth rates. MiR-141-3p, a tumor suppressor miRNA in some cancers such as glioma [144], was found to be endogenously bound by MALAT1, resulting in its inhibition [145]. To understand the role of miR-141-3p in PM, a study highlighted its ability to downregulate YES-associated protein 1 (YAP1) [145] Table 3. YAP is a transcriptional coactivator for the Hippo signaling pathway and is identified as an oncogene [146]. This enabled the discovery of a new regulatory pathway, MALAT1/ miR-141-3p/YAP1 [145] Table 2. It is notable that pemetrexed, one of the very few chemotherapeutic options for PM patients, was found to have an inversely correlated response with MALAT1 [147]. In summary, MALAT1 overexpression in PM has negative effects on cell division, migration and invasion.

MALAT1 was found to be related to tumor origin in a study that measured its levels after tumor resection, revealing a dramatic decrease in its circulating levels. On the other hand, ectopic implantation of tumor xenografts in mice led to a high surge in MALAT1 plasmatic levels. Furthermore, it has been recorded to have a high specificity of 84.8% for the detection of prostate cancer after analysis of its plasma levels [148]. Whole blood was used to analyze MALAT1 levels by qPCR in metastatic lung cancer patients and controls revealing higher levels in metastatic patients [149]. When measured in the plasma of epithelioid PM patients, MALAT1 was found to be upregulated compared to healthy volunteers [63].

#### H19

H19 is a lncRNA encoded by the H19 gene and is one of the first discovered lncRNAs [150]. Its pathological role has been elucidated through its involvement in angiogenesis, as well as, in the development and progression of various cancers as in breast, respiratory, digestive, genitourinary, nervous system and others [151]. H19 overexpression has been documented in many tumor types, leading to the manifestation of various oncogenic behaviors including increasing cell growth, viability, invasion, metastasis, EMT, colony formation, and alterations in glucose metabolism [152–155]. Furthermore, it plays a role in miRNA mediated regulatory pathways, thereby facilitating the downstream cancerous processes [156].

In-silico analysis of H19 expression in PM revealed its overexpression in the sarcomatoid subgroup [80]. Bueno et al. also highlighted the upregulation for H19 in differential analysis between epithelioid versus sarcomatoid subtypes [81]. The overexpression of H19 in PM is associated with worse overall survival [80]. One detrimental effects of H19 expression is the induction of cisplatin chemo resistance in lung cancer cell lines [157] Table 2. Yet, no enough studies were available on H19 and its oncogenic regulatory pathways or its molecular involvement in PM.

H19 exhibits high diagnostic power in gastric cancer when assessed in plasma, outperforming conventional markers for gastric cancer [158]. In a study involving 66 NSCLC patients, H19 plasma levels were evaluated against 31 patients with benign lung diseases as controls. Significant expression was observed in NSCLC patients, with a sensitivity of 67.74%, specificity of 63.08% and AUC of 0.73. This study identified H19 as a potential plasma diagnostic biomarker [159].

#### PCAT6

Prostate Cancer Associated Transcript 6 (PCAT6) is one of the newly discovered carcinogenic lncRNA located on chromosome 1q32.1. Initially identified in prostate cancer. PCAT family consists of 121 lncRNA [160, 161]. Its carcinogenic action was first recognized through its ability to increase proliferation and colony formation in prostate tumor cells. Since its discovery, PCAT6 has been implicated in playing a major oncogenic role in numerous cancers including lung cancer, colorectal cancer, bladder cancer, breast cancer, osteosarcoma and gastric cancer. To date, the precise oncogenic mechanism for PCAT6 in cancer remains largely unknown and is still in its infancy [162].

PCAT6 has been studied in lung cancers, where it was found to be upregulated in NSCLC tissues compared to normal ones [163]. Additionally, its upregulation was observed to be more pronounced in squamous cell carcinoma than in adenocarcinoma [164]. Overexpression of PCAT6 in NSCLC has been linked to an increase in tumor growth rate, migration and invasion, while its knockdown inhibited growth by inducing cell cycle arrest and apoptosis [163]. Furthermore, PCAT6 expression was associated with tumor size, staging, metastasis and low overall survival in lung cancer [165].

Studies on PCAT6 in PM have revealed the overexpression of lysine-specific demethylase 5B (KDM5B), a lysine demethylase with a known association with PCAT6, in all histological subtypes. Another study corroborated these findings, showing that KDM5B is upregulated in 14% of PM cases [80, 166] Table 2. This lysine demethylase has been implicated in inducing EMT in various cancers, including lung cancers [167–169].

The sensitivity and specificity of PCAT6 were noted to be higher than carcinoembryonic antigen (CEA) in NSCLC patients. Thus, plasma expression levels of PCAT6 were measured in 51 lung squamous cell carcinoma patients, 73 lung adenocarcinoma patients, and 39 normal healthy controls. Given its circulation levels in patient’s blood in lung cancer, PCAT6 could potentially serve as a diagnostic and prognostic biomarker in PM, a possibility that requires further explanation [170].

While there is no direct evidence that PCAT6 has diagnostic or prognostic potential in PM, its detectability in plasma of NSCLC patients suggests it as a possible marker for PM. The ability of PCAT6 to be reliably detected and quantified in plasma of NSCLC supports it as a feasible non-invasive blood test. Multiple studies showed that lncRNAs remain stable in plasma and this could be a general principle to be applied for PCAT6 in thoracic cancers as well. Since both PM and NSCLC are thoracic cancers arising from thoracic tissues, they may share overlapping pathogenic mechanisms. Consequently, if PCAT6 is detectable in the plasma of NSCLC, it is suggested to be released as well in other thoracic malignancies such as PM. Furthermore, the cross-cancer expression of lncRNAs highlights that many oncogenic lncRNAs could be detected among different cancer types, not just the one where they were first described. This could be the case with PCAT6 serving as a pan-cancer biomarker.

#### EGFR-AS1

Epidermal growth factor receptor anti sense-1, EGFR-AS1. This lncRNA has been identified as a regulator of EGFR in liver cancer [171]. EGFR-AS1 was shown to be upregulated in PM patients and associated with high resistance to therapy by EGFR tyrosine kinase inhibitor (TYR) [172, 173] Table 2. Knockdown of this lncRNA led to increased sensitivity to EGFR-TYR squamous cell carcinoma [172], suggesting a potential target for PMP treatment. Although, this lncRNA is suggested to play an important role in EMT, its exact role has not yet been determined [80].

#### ZEB2-AS1

ZEB2-AS1, also known as ZEBNAT, has been identified as a regulator of ZEB in EMT induced by transforming growth factor beta (TGF-β) in bladder cancer cells [174]. Knocking down ZEB2-AS1 results in lower expression of N-cadherin and vimentin, and restoration of E-cadherin expression in hepatocellular carcinoma [175]. In an analysis conducted by Lopez-Rios, ZEB2 was found to be associated with the sarcomatoid subtype, which is the most aggressive subtype of PM [176]. Another analysis by Bueno et al. identified significant alterations in the ZEB2 gene between sarcomatoid and epitheloid PM [81]. In-silico analysis revealed dysregulation of ZEB2-AS1 in PM. However, further studies are needed to elucidate its exact pathogenic role in PM and its potential use as a therapeutic target [80].

#### NEAT1

EF177379 or NEAT1 is reported to be involved in mRNA transport regulation and is a crucial component of paraspeckles [177]. It has been demonstrated to regulate the retention of mRNAs containing Alu within the nucleus, which greatly affects gene expression [178].

It is BAP1 dependent and it promotes EMT through regulation of EZH2 [179–181]. BAP1 mutations are present in almost 65% of mesotheliomas Table 2. Consequently, this will harbor implications in the part of NEAT1 in PM [182]. Furthermore, the location of NEAT1, 11q13.1, was reported to be amplified in PM [53].

More studies are needed to examine the role of NEAT1 in PM. However, it has been downregulated in PM. Upon histological stratification after TCGA dataset analysis, epithelioid subtype has the highest levels of NEAT1 [80].

#### HOTAIR

HOX transcript antisense RNA, HOTAIR. This lncRNA is transcribed from the HOXC gene cluster. It is well-known to be overexpressed in various solid malignancies and to play a role in metastasis and tumor recurrence [139, 183]. The role of HOTAIR in EMT is mediated by the regulation of PRC2 and its recruitment to CDH1 promoter [184]. Another mechanism by which HOTAIR regulates EMT is through the expression regulation of JMJD3 and Snail [185]. Moreover, it takes part in the silencing of numerous anti-EMT regulators as miR-7 and miR-34a [186, 187]. Cisplatin resistance has been associated with HOTAIR expression in lung adenocarcinoma due to P21 ^WAF1/CIP1^ [188].

In PM, HOTAIR is overexpressed in the sarcomatoid subtype and associated with poor overall survival [80, 81]. Additionally, in-silico analysis of TCGA dataset revealed HOTAIR overexpression in the biphasic subtype [80].

#### MYCNOS

MYCNOS is a lncRNA that regulates the expression of N-Myc transcription factor, which plays a role in driving EMT [189–191]. Although it is upregulated in some PM patients, with the majority being of the biphasic subtype, its exact role in PM has not yet been identified [80].

#### HULC

Highly Upregulated in Liver Cancer (HULC) was first identified in hepatocellular carcinoma (HCC) to be upregulated [192]. HULC regulations included inhibition for c-Myc expression as well as PI3K/Akt signaling [193]. Furthermore, HULC together with MALAT1 have shown to promote aggressiveness in liver cancer stem cell and act as transcription regulator through cooperation with EZH2 [194, 195]. Concerning the EMT promotion, HULC act as ceRNA for miR-200a-3p and miR-372 [196, 197], to upregulate ZEB1 [196] or Snail [198]. HULC has been overexpressed in epithelioid when compared to sarcomatoid subtype [81]. In-silico analysis showed that 7% of the samples have HULC amplifications or deletions. Yet, exact role of HULC in PM has not been fully understood [80].

#### SNHG7

The small nucleolar RNA host gene 7 (*SNHG7*) or AK054908 is bidirectional lncRNA which encodes for SNORA17 and SNORA43. Such small nucleolar RNAs are crucial players in the post-translational and ribosomal RNA modifications. Generally, Inhibition of ribosome biogenesis, inhibited cell motility, invasion and metastasis. SNHG7 was found to be associated with nodal metastasis and has a potential as prognostic marker in mesothelioma [199]. Yet, upregulation of SNHG7 leads to alteration in ribosomal biogenesis and so cell division needs to be studied [53].

#### AF268386

Long intergenic noncoding RNA AF268386 is involved in myriad of biological processes, including cell cycle regulation and gene regulation via chromatin remodeling [53]. Epigenetic modifications, such as DNA methylation, have been reported in PM. These modifications include fragile histidine triad (*FHIT*; 78%), hypermethylation of E-Cadherin (*ECAD*; 71.4%), *RASSF1A* (19.5%), *RARB* (55.8%), *DAPK* (20%) and the secreted frizzled related protein family (*SFRP*s) [200, 201]. AF268386 demonstrated consistent up-regulation using both microarrays and RT-qPCR when compared to benign pleura [53].

## Discussion

Many lncRNAs have been found to be dysregulated in cancer. They were found to be abundantly distributed in various compartments of the body, especially the tumor microenvironment. The diverse involvement of lncRNAs in cancer initiation and progression, particularly PM, sheds light on their many activities during tumourigenesis. Several lncRNAs, including MALAT1, SNHG8, and POT1-AS1, have been identified as overexpressed in PM tissues and cell lines. Their enhanced expression is associated with cancer-promoting characteristics such as increased invasion, migration, and proliferation, as well as the inhibition of apoptosis. Tumor suppressor lncRNAs, such as CASC2, show lower expression in PM, indicating a lack of regulatory influence over tumor development and progression.

One of the many avenues of personalized medicine involves exploiting biological molecules as therapeutic targets when their role in the pathogenesis of the disease is established. Several compounds are currently in the early stages of drug development as targeting agents for RNA in various cancers [202], including the previously discussed MALAT1 [203].

With oncogenic lncRNAs, inhibition of their biogenesis through various means can be beneficial. The majority of lncRNAs share structural resemblances with mRNA. They are transcribed by RNA polymerase II and RNA polymerase III in eukaryotes, leading to similarities in splicing signals, intron/exon lengths, and typical histone modification profiles. The resulting transcribed lncRNAs are further capped by 7-methyl guanosine at the 5’ end and Poly A tail at the 3’ end, and finally undergo splicing together [204, 205]. However, some lncRNAs lack these features and must be stabilized in other ways, especially at their 3’ ends [206]. There are more nuanced differences in biogenesis that distinguish them from each other. For example, while most lncRNAs are capped, polyadenylated, and spliced by the canonical sites, some lncRNAs such as MALAT1 and Menβ undergo special 3′-terminal processing through alternative processing by endoribonucleases [207].

### Evolving frontline mesothelioma therapies

The treatment landscape has indeed shifted towards immunotherapy-based regimens in unresectable PM; however, this transition is not without considerable academic scrutiny. While trials like CheckMate 743 have shown that Nivolumab + Ipilimumab yielded a median OS of 18.1 months versus 14.1 months for chemotherapy, with an HR of 0.74, *p* = 0.002, the superiority of this regimen is a subject of active debate.

Critical appraisals in the Journal of Thoracic Oncology and JAMA Oncology further put these results in the shadow by emphasizing two significant design limitations:

The first is; histological heterogeneity; the OS benefit was disproportionately driven by non-epithelioid subtypes (HR 0.46) while for the more common epithelioid population, it was far less robust (HR 0.86), raising questions as to whether dual-IO shall be applied as a standard for all patients [25, 208, 209].

Second, suboptimal control arms; the CheckMate 743 study directly compared IO to platinum + pemetrexed alone, and this control arm did not include the anti-VEGFA antibody bevacizumab, which in the prior MAPS study established a survival benefit. The result is that the “IO win” might be less clear-cut if the study is directly compared to the true standard of care globally [26, 210].

Further confusion is added by KEYNOTE-483, in which pembrolizumab with chemotherapy showed an OS advantage with a low HR of 0.74. This provides quick tumor results with increased Objective Response Rate (ORR), but concerns arise with regard to cumulative toxicity in relation to marginal gains in the epithelioid subgroup [211,212]. In relapsed settings, results from PROMISE-meso, published in JAMA Oncology, did not find an advantage in PFS and OS with pembrolizumab compared with chemotherapy, again confirming IO as a target therapy rather than a standard approach [213]. Table 4 highlights the key trial evidence and their corresponding limitations.

**Table 4:** Summary of key trial evidence and critiques

Trial	Reported benefit	Limitation	References
CheckMate 743	Improved OS (18.1 vs. 14.1 months)	Benefit mainly in non-epithelioid; control arm lacked bevacizumab	[25]
PROMISE-meso	Higher response rates (22% vs. 6%)	No difference in PFS or OS compared to chemotherapy	[227]
KEYNOTE-483	Improved OS with chemo + IO	Modest benefit; raises concerns about increased toxicity vs. marginal gain	[224]

There are several ongoing clinical trials for pleural mesothelioma; however, none of these trials explicitly focus on directly targeting lncRNAs. There is a Phase 1 study using Mesomir, a miR-16 mimic, for pleural mesothelioma [214] Table 5, which indicates that RNA-based therapeutics are being explored in the clinical setting for this disease. Additionally, FLM-7523, an antisense oligonucleotide targeting MALAT1, is advancing towards Phase 1 clinical trials for solid tumors [215] Table 5. The ongoing clinical trials for PM primarily focus on immunotherapy, chemotherapy combinations, targeted therapies against specific proteins (like mesothelin or EZH2), and viral therapies [108]. The clinical evaluation of lncRNA-targeting therapies for PM appears to be an emerging area, with the field primarily focused on preclinical investigations at this time.

**Table 5:** Clinical trials evaluating RNA-Based therapies for mesothelioma

Trial Phase		Therapeutic Agent		Target		Brief Description/Objective		Status		References	
Phase 1		Mesomir (miR-16 mimic)		miR-16		Safety of the mimic miRNA agent		Completed		[214]	
Phase 1		FLM-7523 (ASO)		MALAT1		First-in-class inhibitor of MALAT1		Advancing towards Phase 1		[215]	
Preclinical		Small molecules or ASOs		LINC00152–EZH2 interaction		Block binding between LINC00152 and EZH2, so inhibiting oncogenic downstream signaling		Proposed therapeutic strategy		[223]	
Preclinical		Small molecules or ASOs		GAS5–miRNA interactions		Targeting GAS5–miRNA binding sites, preventing oncogenesis-related effects		Proposed therapeutic strategy		[223]	
Preclinical		Small molecules		MALAT1 3’-end triple helix		Disruption of MALAT1 processing by destabilizing its exonuclease-protected 3’-end triple helix to reduce MALAT1 levels		Proposed therapeutic strategy		[223]	

### Modes of targeting LncRNA

**Fig. 2:**
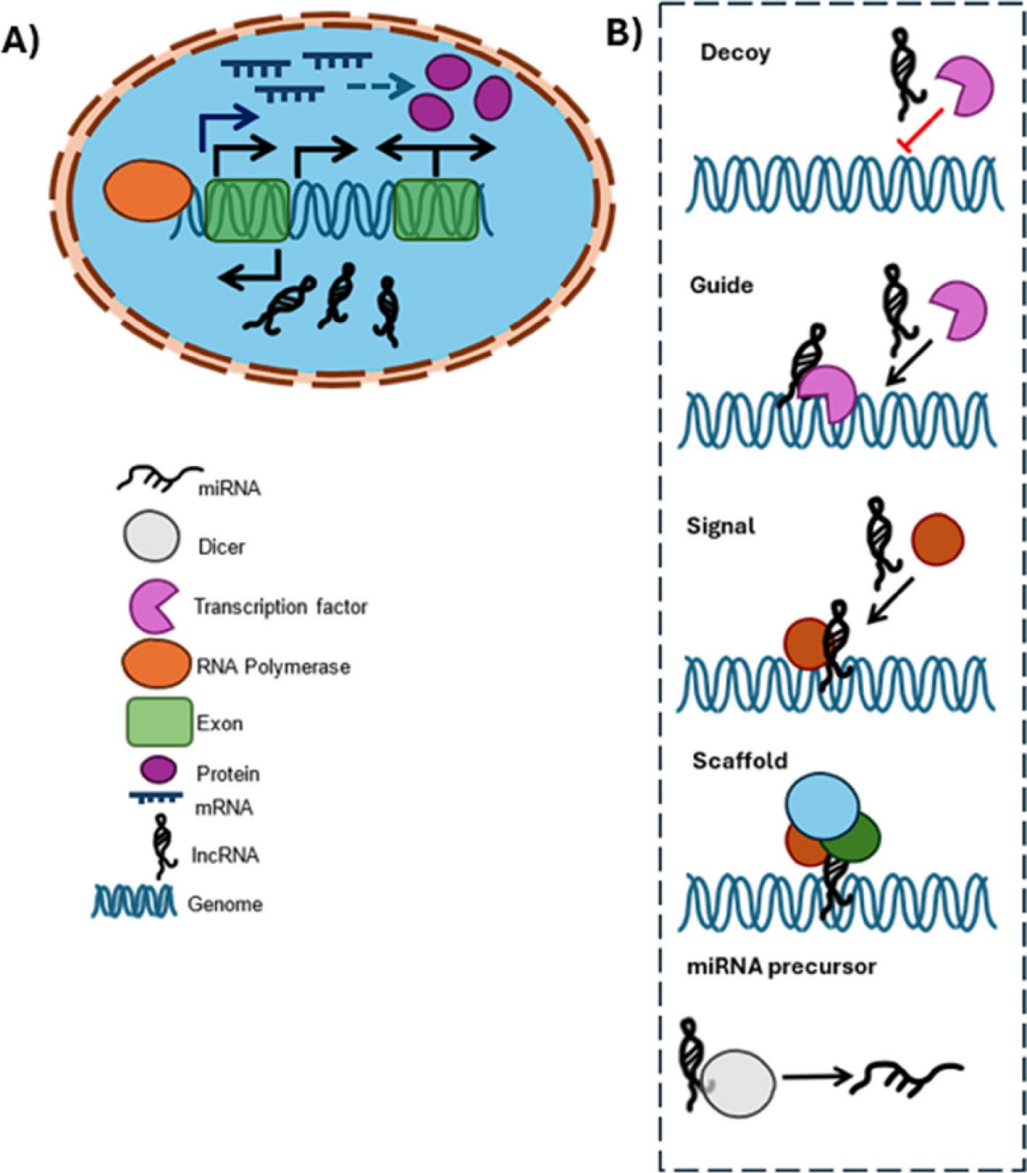
**A** Schematic diagram of the biogenesis of lncRNA with labels. **B** The diverse functions of lncRNA. *Scaffold*: helps form RNA-protein or Protein-Protein complexes which could suppress or activate gene transcription. *Decoy*: Eliminate regulation of genome by binding to transcription factors. *miRNA Precursor*: Act as precursors that are processed into mature miRNA. *Signal*: Act as Signaling molecules that initiate transcriptional activity. *Guide*: Leads Transcription factors and other signaling molecules to their site of binding

Various modes of action can be considered: (i) modulating lncRNA levels transcriptionally or (ii) modifying RNA processing post transcriptionally [216] taking into account the expression process Fig. 2 [40].

To achieve the former, the promoter can be blocked using small molecules or antisense oligonucleotides (ASOs), and morpholinos, or by utilizing silencing techniques such as RNAi or CRISPR/Cas9 to delete relevant lncRNA loci. To achieve the latter, degradation pathways can be triggered through two methods : either by using chemically modified ASOs that trigger RNase H-dependent cleavage mechanisms, leading to cleavage of the target RNA [217], or by using a double stranded siRNA to trigger a pathway involving a RNA-induced silencing complex (RISC).

An alternative method involves using ASOs that act as antagonists to natural antisense lncRNAs (NATs). RNA molecules generated from the opposing strand of protein-coding genes are known as NATs. These NATs fall into two categories: trans-NATs, which affect genes farther down the genome, and cis-NATs, which affect genes nearby [218]. NATs are widely distributed across the human genome, and it appears that their main role is local regulation—specifically, inhibiting the expression of protein-coding genes that overlap with them [219]. This suggests that NATs may function as regulatory elements, acting as repressors of gene expression. They are often found in close proximity to tumor suppressor genes [220] and have been observed to suppress these genes when upregulated [219].

Hence, ASOs can be engineered to inhibit sense-antisense interactions, thereby suppressing the activity of NATs and enhancing tumor suppressor activity.

Regarding tumor suppressor lncRNAs, strategies involving targeted delivery to the tumor site can be employed to exert their effects. Various carrier systems are currently under investigation for delivering ncRNAs [221], and several in-vivo mRNA delivery systems have already advanced to clinical trials [222], potentially paving the way for similar approaches in the future.

A majority of the lncRNAs that have been associated with PM were found to be oncogenic, and hence approaches where interactions between these lncRNAs and their binding partners are disrupted presents a strategic approach to interfere with oncogenic signaling pathways. For example, small molecules or ASOs can be designed to target the binding sites between LINC00152 and EZH2 or between GAS5 and its miRNA targets. By interrupting these interactions, the downstream effects facilitated by these lncRNAs in oncogenesis, such as enhanced cell proliferation and invasion, can be stopped. Another approach that may prove affective in PM is disrupting RNA processing to hinder lncRNA expression. MALAT1 levels, for example, may be reduced through targeting its exonuclease protecting 3’-end triple helix structure (227).

## Conclusion

Treatment of PM using lncRNAs is still in its infancy, and the currently available treatment options often do not yield significant success due to late diagnosis. LncRNAs have been implicated in a myriad of pathological pathways and have been utilized in the treatment of various cancers. However, while lncRNAs play pivotal roles in the treatment of many cancers, their roles in PM have not been fully elucidated, and their use in treatment remains unexplored. long non-coding RNAs are increasingly recognized as important players in the pathogenesis of PM, with several lncRNAs showing dysregulated expression and functional involvement in key oncogenic processes. Preclinical studies have provided compelling evidence that targeting specific lncRNAs, such as PVT1, MALAT1, and LINC00152, in vitro can inhibit the proliferation, migration, and invasion of PM cells.

Innovative therapeutic methodologies can also be developed by exploring the pathophysiology of PM and the lncRNAs involved, with a growing body of evidence highlighting them as regulators in the tumor microenvironment. One example of a feature is hypoxia. While solid tumors may be present in both oxygenated and hypoxic environments, tumor hypoxia has been associated with poor prognosis and increased resistance in chemotherapy and radiation therapy [228]. Hypoxic reactions are primarily regulated by transcription factor hypoxia-inducible factor (HIF) as a method of modulating metabolism through the reduction of oxygen consumption. HIF and its subunits have been shown to undergo lncRNA based regulation [74, 229–231] through various diverse mechanisms at all stages of expression [232]. PM based tumors have been showed to display large hypoxic areas [233] which adds an additional layer of limitations to pre-existing traditional therapies which may be overcome by the incorporation of lncRNA in future strategies.

While direct clinical trials evaluating lncRNA-targeting therapies for PM are still in their nascent stages, the ongoing exploration of miRNA-based therapeutics and the advancement of MALAT1 inhibitors signal a growing interest in the potential of RNA-based interventions for this challenging disease. This article sheds light on the lncRNAs tied to PM pathophysiology whether directly or indirectly thus far, which could hold therapeutic potential.

## Data Availability

No datasets were generated or analysed during the current study.

## Abbreviations

PM: Pleural mesothelioma
ASOs: Antisense oligonucleotides
AUC: Area under the curve
BCL2L14: Bcl-2 like protein 14
BIRC8: Baculoviral IAP repeat-containing protein 8
CASC2: Cancer susceptibility candidate 2
CEA: Carcinoembryonic antigen
EMT: Epithelial-mesenchymal transition
EPP: Extrapleural pneumonectomy
EZH2: Enhancer of zeste homolog 2
FASLG: FS ligand
FOXM1: Forkhead box M1
FRS2: Fibroblast growth factor receptor substrate 2
GAS5: Growth arrest-specific transcript 5
HCC: Hepatocellular carcinoma
HULC: Highly Upregulated in Liver Cancer
IL-24: Interleukin-24
IMRT: Intensity modulated radiotherapy
IO: Immuno-Oncology Therapies
KDM5B: Lysine-specific demethylase 5B
LINC00689: Long Intergenic Non-Protein Coding RNA 689
LncRNA: Long non-coding RNA
LTB: Lymphotoxin beta
LUAD: Lung adenocarcinoma
MALAT1: Metastasis-associated lung adenocarcinoma transcript 1
NAT: Natural antisense
NSCLC: Non-small cell lung cancer
OS: Overall Survival
P/D: Pleurectomy/decortication
PCAT6: Prostate Cancer Associated Transcript 6
PVT1: Plasmacytoma variant translocation 1
RISC: RNA-induced silencing complex
ROC: Receiver operating characteristic curve
siRNA: Short interfering RNA
SNHG7: Small nucleolar RNA host gene 7
SOX2: SRY-box2
TCGA: The Cancer Genome Atlas
TGF-β: Transforming growth factor beta
TNFRSF1B: Tumor necrosis factor receptor superfamily member 1B
TYR: Tyrosine kinase inhibitor
YAP1: YES-associated protein 1
